# Neutrophilic Dermatoses and Their Implication in Pathophysiology of Asthma and Other Respiratory Comorbidities: A Narrative Review

**DOI:** 10.1155/2019/7315274

**Published:** 2019-06-10

**Authors:** Iman Salem, Mark Kimak, Rosalynn Conic, Nicola L. Bragazzi, Abdulla Watad, Mohammad Adawi, Charlie Bridgewood, Alessia Pacifico, Pierachille Santus, Maurizio Rizzi, Stephen Petrou, Delia Colombo, Marco Fiore, Paolo D. M. Pigatto, Giovanni Damiani

**Affiliations:** ^1^Department of Dermatology, Case Western Reserve University, Cleveland, USA; ^2^Department of Health Sciences (DISSAL), School of Public Health, University of Genoa, Genoa, Italy; ^3^Department of Medicine “B”, Zabludowicz Center for Autoimmune Diseases, Sheba Medical Center, Tel-Hashomer, Israel; ^4^Sackler Faculty of Medicine, Tel-Aviv University, Tel-Aviv, Israel; ^5^Section of Musculoskeletal Disease, Leeds Institute of Molecular Medicine, University of Leeds, NIHR Leeds Musculoskeletal Biomedical Research Unit, Chapel Allerton Hospital, Leeds, UK; ^6^Padeh and Ziv Hospitals, Azrieli Faculty of Medicine, Bar-Ilan University, Ramat Gan, Israel; ^7^San Gallicano Dermatological Institute, IRCCS, Rome, Italy; ^8^Department of Biomedical Sciences L. Sacco, University of Milan, Milan, Italy; ^9^Respiratory Unit, Center for Sleep and Respiratory Disorders, “Luigi Sacco” University Hospital, Milan, Italy; ^10^Emergency Medicine, Good Samaritan Hospital Medical Center, New York, USA; ^11^Department of Pharmacology, University of Milan, Milan, Italy; ^12^Department of Women, Child and General and Specialized Surgery, University of Campania “Luigi Vanvitelli”, Naples, Italy; ^13^Clinical Dermatology, IRCCS Galeazzi Orthopaedic Institute, Milan, Italy; ^14^Department of Biomedical, Surgical and Dental Sciences, University of Milan, Milan, Italy; ^15^Young Dermatologists Italian Network, Centro Studi GISED, Bergamo, Italy; ^16^Dipartimento di Fisiopatologia Medico-Chirurgica e dei Trapianti, Università degli Studi di Milano, Unità Operativa di Dermatologia, IRCCS Fondazione Ca' Granda, Ospedale Maggiore Policlinico, Milan, Italy

## Abstract

Neutrophilic dermatoses (ND) are a polymorphous group of noncontagious dermatological disorders that share the common histological feature of a sterile cutaneous infiltration of mature neutrophils. Clinical manifestations can vary from nodules, pustules, and bulla to erosions and ulcerations. The etiopathogenesis of neutrophilic dermatoses has continuously evolved. Accumulating genetic, clinical, and histological evidence point to NDs being classified in the spectrum of autoinflammatory conditions. However, unlike the monogenic autoinflammatory syndromes where a clear multiple change in the inflammasome structure/function is demonstrated, NDs display several proinflammatory abnormalities, mainly driven by IL-1, IL-17, and tumor necrosis factor-alpha (TNF-a). Additionally, because of the frequent association with extracutaneous manifestations where neutrophils seem to play a crucial role, it was plausible also to consider NDs as a cutaneous presentation of a systemic neutrophilic condition. Neutrophilic dermatoses are more frequently recognized in association with respiratory disorders than by chance alone. The combination of the two, particularly in the context of their overlapping immune responses mediated primarily by neutrophils, raises the likelihood of a common neutrophilic systemic disease or an aberrant innate immunity disorder. Associated respiratory conditions can serve as a trigger or may develop or be exacerbated secondary to the uncontrolled skin disorder. Physicians should be aware of the possible pulmonary comorbidities and apply this knowledge in the three steps of patients' management, work-up, diagnosis, and treatment. In this review, we attempt to unravel the pathophysiological mechanisms of this association and also present some evidence for the role of targeted therapy in the treatment of both conditions.

## 1. Introduction

The idea of a greater understanding of how body organs operate in interconnected ways to influence the homeostatic capacities of one another is gaining attention in modern medicine [1]. One such example arises from the physiological and pathological relationship between the skin and respiratory systems. Continual accumulating evidence supports the idea that these two systems are uniquely related in function [2]. As a primary interface with the outside world, both are equipped with specialized immune defenses and colonized by microbial commensals, crucial factors in regulating and maintaining homeostasis. Additionally, western medicine has observed interesting associations of some chronic inflammatory dermatoses with both allergic and nonallergic respiratory conditions [3, 4]. In this discussion we investigate the relationship between neutrophilic dermatoses and respiratory disorders in order to demonstrate the impact of neutrophils in this associated comorbidity. We also attempt to shed light on potential strategies for optimizing the management of both conditions [5].

## 2. Neutrophilic Dermatoses

Neutrophilic dermatoses (ND) are a polymorphous group of noncontagious dermatological disorders that share the common histological feature of a sterile cutaneous infiltration with mature normal neutrophils [6, 7]. Clinical manifestations can vary from nodules, pustules, and bulla to erosions and ulcerations. Furthermore, NDs can be histologically subcategorized according to predominance of the neutrophilic infiltrate in different skin layers into (1) epidermal NDs as seen in pustular psoriasis, IgA pemphigus, acute generalized exanthematous pustulosis (AGEP), a microbial pustulosis of the folds, and a microbial pustulosis of the scalp; (2) dermal NDs as in acute febrile neutrophilic dermatosis or Sweet's syndrome (SS), erythema elevatum diutinum, and neutrophil eccrine hidradenitis; and (3) dermo-hypodermal NDs as in hidradenitis suppurativa (HS), neutrophilic panniculitis, aseptic abscesses syndrome, and pyoderma gangrenosum (PG). Despite the fact that the etiology of ND is often idiopathic, interesting associations have been found with other systemic preceding illnesses. Common examples include the frequent finding of Sweet syndrome with myeloid hematologic malignancies and the eruption of pyoderma gangrenosum in patients with rheumatoid arthritis and inflammatory bowel disease, separately and sometimes together. Similarly, cases of erythema elevatum diutinum and subcorneal pustular dermatosis were reported with monoclonal gammopathies (erythema elevatum diutinum, subcorneal pustular dermatosis). These observations draw attention to a possible infiltration of interior organs with neutrophils during the establishment of ND, thus promoting the notion of neutrophilic systemic disease [8, 9]. The etiopathogenesis of NDs is still debatable. NDs do indeed share some pathogenetic and clinical features with autoinflammatory diseases, defined as conditions that manifest themselves as recurrent episodes of inflammation in targeted organs, without evidence of infection, high titer of circulating autoantibodies, and T autoreactive cells, unlike the monogenic autoinflammatory syndromes where a clear alteration in the inflammasome structure/function is demonstrated. NDs display several proinflammatory alterations mainly driven by IL-1, IL-17, and tumor necrosis factor-alpha (TNF-a); however these remain an important matter for discussion, mainly because of their rarity and difficulties in diagnosing. What remains certain is the role of dysregulated PMNs homeostasis in the development of cutaneous and extracutaneous manifestations in NDs [10–13]. The main NDs with their respiratory comorbidities are summarized in Table 1.

### 2.1. The Role of Neutrophils in the Pathogenesis of Neutrophilic Dermatoses

Neutrophils represent the main component of white blood cells (WBCs) and constitute the first line of defense against external as well as internal insults. During acute inflammation, neutrophils are the first immune cells to migrate from the blood to the site of inflammation site by chemotaxis. Elimination of pathogens occurs by means of phagocytosis, formation of neutrophil extracellular traps (NETs), and the production of antimicrobial granules. Furthermore, neutrophils are crucial to the expansion and provocation of inflammatory responses through the release of chemokines and cytokines resulting in the recruitment and activation of more neutrophils in addition to other innate and adaptive immune cells such as macrophages and natural killer cells, T helper type 1 (Th1) and Th17 cells [14].

Although this progression is necessary to mount an effective immune reaction aimed at clearing invaders, the accompanied inflammation is often detrimental to the host cells and can end in serious pathological conditions because of the uncontrolled, persistent activation of neutrophils and the innate response. This can lead to a more chronic inflammation and ultimately tissue destruction. Therefore, tight regulation of neutrophil migration and activation is pivotal [14]. Recently neutrophilic dermatoses have been linked to the concept of autoinflammation. Mounting evidence of a common cytokine profile including IL-1 beta, TNF, and IL-17 in the lack of any evidence of allergy, infection, or autoimmunity has supported this argument. In addition, the identification of multiple overlapping genetic, clinical and histologic features with autoinflammatory conditions further validates this theory. Additionally, the frequent association of NDs with systemic inflammatory diseases where neutrophils seem to play a prominent role promotes the idea of NDs being a polygenic cutaneous autoinflammatory condition with a potential neutrophilic infiltration of interior organs resulting in a neutrophilic systemic disorder [14–16]. In support of this hypothesis was the efficacy of interleukin-1 (IL-1) blocking therapeutic agents in the management of some neutrophilic dermatoses [9]. Being aware of this connection will provide new insights into the treatment of refractory comorbidities encountered with neutrophilic dermatoses. Inflammasomes are understood as fundamental in the pathogenesis of autoinflammatory disease. These cytoplasmic proteins critically regulate the proteolytic cleavage and activation of IL-1 beta and IL-18, strong proinflammatory cytokines. Activation of IL-1 beta production requires two signals. The first is a priming signal to induce the expression of the pro-IL-1, occurring as a result of the activation of myeloid differentiation gene 88 (MyD88) dependent nuclear factor (NF)-KB mediated by stimulation of Toll like receptors (TLRs), and then full activation of inflammasome complex is achieved when its cytoplasmic pattern recognition receptors receive either damage or pattern associated molecular pattern signals. As a result, caspase-1 is activated which in turn processes pro-IL-1 into functional IL-1. Once formed, active IL-1 signaling then amplifies neutrophil mediated inflammation through multiple mechanisms. Further, IL-1b has antiapoptotic effects on neutrophils prolonging their survival; neutrophils in turn increase the activation of Pro-IL-1 to mature IL-1b in both inflammasome dependent (caspase-1 dependent) and/or inflammasome independent manner through the release of elastase, proteinase-3, and cathepsin G serine proteases. Additionally, enhancement of neutrophil recruitment and activation can occur directly and indirectly through IL-1 driving Th17 immune response [10, 14].

#### 2.1.1. Association of Neutrophilic Dermatoses with Respiratory Comorbidities

The link between neutrophilic dermatoses and respiratory comorbidities, particularly in the context of their overlapping immune responses mediated primarily by neutrophils, raises the likelihood of a common neutrophilic systemic disease or an aberrant innate immunity disorder. The following observations are representative of the importance of neutrophilic dermatoses in lung-associated clinical presentations and how this can be used to optimize patients' management.

### 2.2. Hidradenitis Suppurativa and Associated Respiratory Comorbidities

#### 2.2.1. Hidradenitis Suppurativa (HS)

Hidradenitis suppurativa (HS) is a rare chronic inflammatory disease typically affecting hair follicles often with a long delay before diagnosis [17]. In order to overcome the delay, several questionnaires were developed such as the visual aided questionnaire that includes a group of specific questions and lesion images [18]. HS is characterized by recurrent formation of multiple abscesses and draining sinuses in apocrine, commonly inguinal, anogenital, and axillary areas, and is therefore referred to as* acne inversa*. Eventually these lesions heal with scarring and disfigurement. Recent evidence has shown HS extending beyond being a disfiguring dermatosis and rather a systemic inflammatory disease with predominant cutaneous presentation [19, 20]. Furthermore, a scoring system, named Autoinflammatory Disease Damage Index (ADDI), capable of accurately evaluating inflammatory-related comorbidities has been also proposed [21].

Hyperkeratosis induced occlusions, and subsequent inflammation of the pilosebaceous unit, together with the dysregulated innate and adaptive immune responses, is a key feature in the pathogenesis of HS. Occluded hair follicles eventually rupture, spilling their contents, including bacteria and keratin debris, into the surrounding dermal tissue resulting in the chemotaxis of neutrophils and lymphocytes. The recruited cellular infiltrate results in abscess formation and destruction of the hair follicles and the surrounding adnexal structures [22]. The role of the Notch signaling pathway in the pathogenesis of HS was recently investigated by Melnik and Plewig in 2013 [23]. A defect found in the Notch pathway thought to be necessary for maintaining the structure of the hair follicle appendage results in the alteration of toll like receptor signaling inducing the release of proinflammatory cytokines, such as tumor necrosis factor (TNF)-*α*, interleukin (IL)-1*β*, and IL-17, thus promoting a Th17 mediated immune response [10, 23].

#### 2.2.2. Association of HS with Neutrophilic Asthma and COPD

The importance of increased airway neutrophil infiltration has long been identified as a cardinal feature of a number of inflammatory pulmonary conditions such as COPD, interstitial pneumonia [24], and asthma, especially severe forms exacerbated by cigarette smoking [25].

A cross-sectional study performed by Magun et al., using Clalit Health Services database (CHS), investigated the frequency of pulmonary diseases in 3207 patients with HS compared to 6412 matched controls. The authors reported a significant association of HS with asthma and chronic obstructive pulmonary disease (COPD) which could be explained by pulmonary neutrophil infiltration metastasizing from skin conditions triggered by genetic and environmental factors known to enhance neutrophil chemotaxis and increase their survival in the airways [26]. Furthermore, increased levels of the proinflammatory cytokines such as interleukin (IL)-17 and tumor necrosis factor (TNF)-*α* could directly contribute to airway inflammation [17].* No correlation is actually demonstrated between HS severity and respiratory involvement.*

#### 2.2.3. Role of Neutrophils in the Pathogenesis of Asthma

Neutrophils have also been linked to a subset of asthma referred to as neutrophilic asthma which is often unresponsive to steroid treatment. In 2010, Uddin et al. correlated the severity of asthma to the neutrophil burden within the airways secondary to the presence of antiapoptotic factors increasing the survival of polymorphonuclear leukocytes in the airway [27]. Baines et al. showed increased neutrophil chemotaxis and survival in noneosinophilic asthma, a type commonly populated with* Moraxella catarrhalis* or* Haemophilus influenzae. H. influenzae*, in particular, is believed to drive Th17-mediated development of neutrophilic asthma through the increase of IL-17 production [28]. IL-17 levels are directly correlated with a rise in IL-8 and other neutrophil chemotactic factors. This cascade is exacerbated further by smoking and other environmental antigens which also act as triggering factors for HS too [29]. Infiltrating neutrophils subsequently produce reactive oxygen species (ROS), IL-33, and TSLP (thymic stromal lymphopoietin), which further perpetuate airway inflammation. Additionally, allergens induce the release of matrix metalloproteinase 9 (MMP9) and chemokines as CXCL8 through the activation of toll like receptors 7 and 8 (TLR-7&8). Their release can in turn recruit more neutrophils in a positive feedback manner. Although clearance of inflammatory cells through programmed cell death and removal of cellular debris are a perquisite step of healing, inappropriate neutrophilic necrosis or the failure of apoptosis may instead enhance ongoing inflammation. Further, some environmental antigens such as house dust mites (HDM-DP) induce the production of myeloid-related protein 8 and 14 which in turn stimulate TLR4/Lyn/PI3K/Akt/ERK/NF-kappaB pathway resulting in increased neutrophil survival due to apoptosis inhibition [29].

#### 2.2.4. Role of Neutrophils in the Pathogenesis of COPD

According to World Health Organization (WHO), chronic obstructive pulmonary disease (COPD) was reported as the fifth leading cause of death globally, carrying a substantial economic and health burden [30]. While the role of neutrophils is confined to certain subtypes of asthma, their contribution in COPD is crucial to disease pathogenesis and the degree of their activation is directly related to the severity of the clinical outcome. For example, increased neutrophil lymphocyte ratio is a reliable predictor of a potential exacerbation. In COPD, neutrophil released proteases are believed to increase pathogen replication through the impairment of the IL-22/IL22R defense mechanism and downregulation of effector antimicrobial molecules like *β*defensin-2, ultimately resulting in acute progression. Additionally, the breakdown of lung elastin by neutrophil derived elastase directly contributes to the inflammatory process of COPD by diminishing the lung's ability to tolerate inflation sufficiently. Additionally, tumor necrosis factor-alpha (TNF-*α*) released by neutrophils upregulates the expression of epidermal growth factor receptors on airway epithelial goblet cell, resulting in mucus hypersecretion thereby further exacerbating airway obstruction. Moreover, the reactive oxygen species and elastases produced by the neutrophils activate the pro-ligand transforming growth factor (TGF) [31].

Cigarette smoking (CS) further worsens the prognosis in both HS and COPD by increasing the release of damage-associated molecular patterns (DAMPs) which, upon binding to TLR 2, 4, and 9, enhance neutrophil recruitment and activation and decrease apoptosis. However, this increase in neutrophilic infiltration is not accompanied by elimination of the pathogen as might be expected. CS results in defective phagocytic activity by the means of inhibition of caspase-3, which ultimately results in persistent infection and delayed healing [29].

#### 2.2.5. Implications of This Association on Treatment Strategies

**Figure 1:**
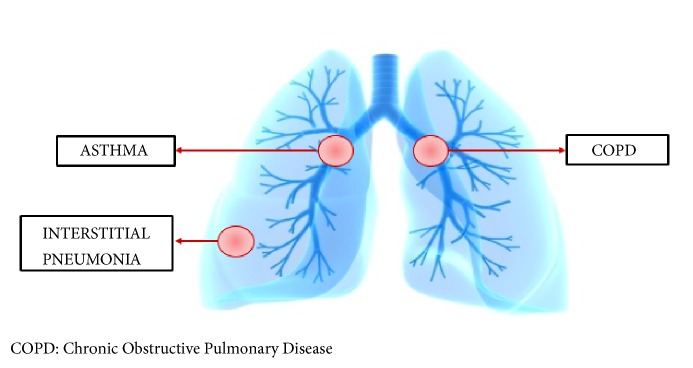
Hidradenitis suppurativa and its described respiratory comorbidities.

In the context of the aforementioned findings, it is plausible to link nonresponsive cases of HS and COPD to the concept of failed removal of excess detrimental neutrophils and that neutrophilic targeted therapy could treat both conditions simultaneously. Consistent with this hypothesis are the findings reported by Tian et al., in 2017. This paper stressed the importance of the granulocyte (neutrophil and eosinophils) apoptosis and clearance for successful elimination of allergen driven airway inflammation among those with steroid-resistant inflammatory respiratory illness [32]. This may be further supported by the observation of improved asthma symptoms with SCH527132, a selective CXCR2 receptor blocker that decreases neutrophils infiltration [33]. Similarly, Anakinra, an IL-1 receptor antagonist, has shown promising results in the treatment of HS. An open-label study conducted by Leslie et al. in 2014 involved the treatment of 6 HS patients with Anakinra for 8 weeks followed by observation of their lesions for another 8 weeks. Results revealed an improvement of HS lesions in all the participants who completed the eight-week trial [34]. Another randomized study performed by Tzanetakou et al. two years later compared the disease activity in 20 participants with HS who were assigned at random to a daily injection of either Anakinra or placebo. The group treated with Anakinra showed decreased disease activity and prolonged remission relative to placebo [35]. Pathogenesis-tailored therapeutics were also investigated in the treatment of COPD. In 2004, Mahler et al. studied the effect of neutralizing IL-8, a strong neutrophil chemotactic factor, with a monoclonal antibody. The researchers reported a symptomatic improvement of COPD in the treated group compared to placebo [36]. Similarly, leukotriene B4 inhibitors and TNF-alpha blockers have shown promising results in halting COPD progression [37].* HS lung comorbidities are summarized in Figure 1.*

### 2.3. Pyoderma Gangrenosum (PG) and Associated Respiratory Comorbidities

#### 2.3.1. Pyoderma Gangrenosum

Pyoderma gangrenosum is a rare inflammatory neutrophilic dermatosis most commonly presenting with a disfiguring deep necrotic laceration with a dusky, indeterminate edge, typically in the lower limb. In addition to the classic ulcerative presentation, four other variants are identified: pustular, bulbous, peristomal, and vegetative [38–41]. Since neither the clinical nor histological manifestations are pathognomonic, PG is usually diagnosed by a combination of features only after exclusion of other conditions [16].

The pathogenesis of PG is complex and not yet fully understood. Its foundational mechanism is believed to be because of a pathological uncontrolled sterile neutrophilic infiltration of the skin, and presumably other organs, in a genetically predisposed individual [42]. Additionally accumulating data support the role of autoinflammation in disease development. Aberrant signals involved in neutrophil trafficking, chemotaxis, metabolic activity, and phagocytic function are among the main driving mechanisms of PG. This possibly explains its common association with other dysfunctional neutrophil-driven systemic disorders. Overexpression of neutrophil chemotactic factors such as IL-8 in cutaneous lesions further consolidates the idea of neutrophils playing a key role in PG. Additionally, PG lesions show high levels of MMP, TNF-alpha, and IL-17. Further favoring the idea of autoinflammation as the nidus of PG comes with the discovery of proline-serine-threonine-phosphatase interactive protein (PSTPIP-1) mutation. This was recently identified in PG-associated syndromes, namely, PAPA (pyogenic arthritis, pyoderma gangrenosum, and acne) and PAPASH (pyogenic arthritis, pyoderma gangrenosum, acne, and hidradenitis suppurativa), each of which includes PG as well as at least one other neutrophilic systemic disorder. Normally the CD2-binding protein encoded by the PSTPIP-1 gene binds pyrin resulting in suppressed inflammasome activation and proinflammatory cytokine production. This loss of function mutation therefore releases the inflammasome from its default inhibition leading to autoactivation of a caspase-1 cascade and triggering a vicious cycle of IL-1*β* overproduction and enhanced neutrophil recruitment and activation. Further supporting the autoinflammation hypothesis is the recognition of upregulation of IL-1*β* and its receptors in these cutaneous eruptions [43, 44].

#### 2.3.2. PG and Extracutaneous Respiratory Involvement

**Figure 2:**
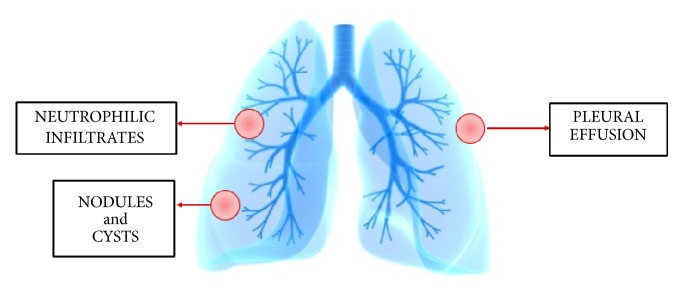
Respiratory comorbidities of pyoderma gangrenosum.

While the exact etiology of PG remains idiopathic, its occurrence is frequently associated with other serious chronic inflammatory conditions, such as inflammatory bowel disease, hematological malignancy, and rheumatoid arthritis, and therefore physicians should maintain vigilance looking for them when a case of PG is diagnosed [39]. Among the rare yet significant extracutaneous manifestations of PG are those involving the respiratory system. Till 2018, only forty-one cases of this comorbidity were described in the international literature [45]. Cutaneous lesions usually precede the pulmonary manifestation, which is sometimes diagnosed several years later after excluding other diagnoses such as lung cancer and Wegner's granulomatosis. Symptoms observed range from cough, dyspnea, hemoptysis, and low grade fever to stridor and fulminant alveolar hemorrhage. Findings on chest X-rays and CT scans of most of these patients demonstrate unilateral or bilateral nodular infiltrates sometimes progressed to cavitation with no predilection of specific lung zone of location. These are sometimes mistaken for lung abscess. Additionally, opacities and pleural effusions were reported as well. The predominant histological feature was neutrophilic infiltration which is in agreement with the neutrophil being the main culprit in the extra cutaneous manifestations. Lesions in the lung, however, rarely show necrotizing granulomas or vasculitis. Additionally, overexpression of IL-17 was recorded in both cutaneous and pulmonary conditions. IL-17 stimulates the production of IL-1*β* and TNF-*α* from macrophages and induces the production of neutrophil chemoattractants from human bronchial epithelial cells [46, 47]. Furthermore, a dramatic improvement of the pulmonary condition was reported after the proper treatment of PG with high doses of steroids or immunosuppressants, which further supports the hypothesis. Therefore physicians should be aware of these* well-established* associations and perform a thorough examinations for every patient with PG to rule out comorbidities [39, 47].* PG respiratory comorbidities are resumed in Figure 2.*

#### 2.3.3. Implications of This Association on Treatment Strategies

Physicians usually follow a stepwise approach in managing PG with the mainstay therapy being immune suppressants such as ciclosporin and corticosteroids, and the choice among them will usually be decided based on local experience. However, promising data on newer targeted treatment such as TNF‐*α* inhibitors, IL-1 beta, and IL-8 blocking agents continue to be reported; this is particularly true for refractory cases not responding to conventional therapy [38]. In an open-label study conducted by Kolios et al., 2015, treatment of five patients with PG lesions resistant to steroids with canakinumab (monoclonal antibody against IL‐1*β*) resulted in complete resolution in three patients and partial improvement in one patient after 16 weeks of treatment [48]. Additionally, in 2017, three recalcitrant cases with PG and rheumatoid arthritis or systemic lupus erythematosus were successfully treated with Anakinra, IL-1 receptor antagonist [40]

### 2.4. Sweet Syndrome and Respiratory Comorbidities

#### 2.4.1. Sweet Syndrome (Acute Febrile Neutrophilic Dermatoses)

Sweet's syndrome, also referred to as acute febrile neutrophilic dermatosis, was first introduced by Dr. Robert Douglas Sweet in the British Journal of Dermatology in 1964 [49]. Sweet syndrome can be divided based on association(s) or preceding event(s) into three subtypes: (a) Classical Sweet syndrome (b) Malignancy-associated Sweet syndrome (c) Drug-induced Sweet syndrome [50, 51]. The syndrome's major defining criteria are based on the typical clinical and histological features required to make the diagnosis in all types, along with two additional minor findings [50] (Table 2). Rash is often preceded by bacterial upper respiratory infection, especially of streptococcal origin [50, 52]. This may be explained by either a hypersensitive reaction to the microbial antigen or a consequence of bacterial-induced overproduction of granulocyte-macrophage colony-stimulating factor (GM-CSF) and granulocyte colony-stimulating factor (G-CSF). This outcome is further supported by the reported development of Sweet syndrome following treatment with these cytokines (GM-CSF, and G-CSF). The implication of a perfunctory bacterial relationship should raise awareness of the importance of first treating the bacterial infection with antibiotics before rushing into the use of corticosteroids with potential side effects [53, 54]. In addition, Sweet syndrome sometimes develops after the use of certain medications, such as all transretinoic acids, adalimumab, ipilimumab, vemurafenib, azathioprine, and some antibiotics. To make a drug-induced diagnosis, the presenting rash should be reproducible with the recurrent use of the same drug and spontaneously resolve upon its withdrawal [50, 55–57] (Wallach and Vignon-Pennamen, 2015, and Vashisht and Holmes, 2017).

Pathogenesis of Sweet syndrome is still not fully delineated. It has been related previously to altered immune response or possibly hypersensitivity to microbial or neoplastic antigens [52]. Recently, a potential link to autoinflammatory underpinnings has gained support through various genetic and molecular discoveries. Modified transcription or function of genes involved in the regulatory signaling pathway of hematopoietic stem cells, such as tyrosine protein phosphatase nonreceptor type 6 gene (PTPN6), has been described. This possibly explained paraneoplastic associations of Sweet syndrome with hematogenous malignancies and/or chronic inflammatory conditions [14, 58]. Furthermore, overexpression of IL-1B, master of the proinflammatory cytokines, along with neutrophilic chemotactic factor, IL-8, is described in Sweet cutaneous and extracutaneous lesions. Also of note, the differential levels of their expression correlate with the various levels of severity observed not only across the spectrum of neutrophilic dermatoses but also on a case to case basis [14, 55, 59].* Pulmonary findings seem to be related mainly to the classical and malignancy-associated Sweet syndrome and usually follow cutaneous manifestations.*

#### 2.4.2. Sweet Syndrome and Pulmonary Neutrophilic Infiltrates

From 1981 to 2015, forty-three cases of Sweet syndrome-associated pulmonary involvement were reported in the literature, of which 11 had exclusively respiratory extracutaneous manifestations. Notably, the other 31 also suffered from multiple other comorbidities. Sweet's rash preceded lung symptoms in 6 cases, while the other 5 of 11 patients complained of the respiratory condition first. Imaging work most frequently revealed interstitial lung opacities that can be unilateral [60–72] or bilateral [73–84] with or without pleural effusions and no site or lobe predilection [78, 85, 86]. A presenting scenario such as this can be easily confused with pneumonia especially when a biopsy from the lesions is not feasible in critically ill patients, those prone to bleeding or other high risk complications [72, 87]. Sampling from skin and lung lesions reveals sterile polymorphonuclear infiltrates [60–62, 68, 70, 71, 73–75, 77, 79, 82–85, 88] in most of the cases, further indicating the role of systemic neutrophil contribution in the development of extracutaneous manifestations in the lung and elsewhere (Takimoto et al., 1991, Yang et al., 2015, Syed et al., 2018) [61, 72, 87]. Outcomes of Sweet-associated pulmonary conditions vary broadly from rapid improvement with corticosteroid treatment to serious pulmonary compromise and respiratory failure. In 1991 Takimoto et al. described a case with repeated episodes of acute febrile neutrophilic dermatosis with interstitial nodular pulmonary infiltrates who at first responded to steroid therapy but became progressively resistant to steroid and ultimately died from respiratory complications [61]. A similar outcome was recorded in 4 other cases [70, 71, 77, 79]. In the context of the evidence presented, even though lung involvement has been rarely reported with Sweet syndrome, the potential development of life-threatening pulmonary complications should be taken into consideration particularly after pneumonia is excluded and antimicrobial treatments have failed [61, 87].

At least 3 additional cases were reported subsequently [51, 87, 89], including a case of acute neutrophilic dermatosis developed in a patient with pulmonary tuberculosis [51]. In view of the above, a crucial distinction should be made as to whether the cutaneous eruption is secondary to the mycobacterial infection or is a primary skin condition with pulmonary symptoms. In the scenario where the lung infection is the primary established diagnosis, systemic steroid should be used with caution to avoid the risk of dissemination of a localized tuberculosis or the activation of a latent infection. Colchicine can be used as an effective and safe substitute [51].

#### 2.4.3. Sweet Syndrome and Lung Cancer

**Figure 3:**
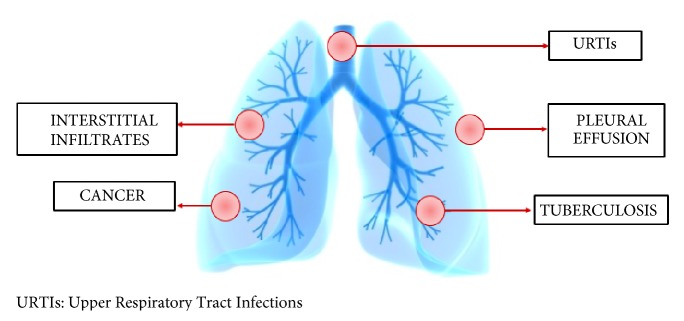
Respiratory manifestations of Sweet syndrome.

Sweet syndrome is frequently described in association with hematogenous malignancy such as chronic myeloid leukemia and lymphoma [50, 54, 90–96]; because of this frequent finding many scientists deem it a paraneoplastic phenomenon [92]. However, while this association is well established for liquid tumors, it is rare with solid tumors including lung cancer. Only seven cases were published describing an association between Sweet syndrome and lung cancer [95]. The first case with lung adenocarcinoma was described by Nielsen et al. in 1993 [92] and one year later Yamamoto et al. identified the first case with small cell lung cancer [93]. It was postulated that paraneoplastic production of IL-8, G-CSF, and GM-CSF by malignant cells was responsible for the extensive recruitment of PMN leukocytes to the skin, accounting for the development of Sweet's syndrome [97]. Moreover, with the progressive increase in both the incidence and annual deaths from lung cancer, much research is dedicated to fully unravel the pathogenesis of this neoplasm, which may lead to a greater appreciation of the role of neutrophils in this condition. In addition, animal studies as well as Bronchoalveolar lavage (BAL) from patients with lung cancer reveal an upregulation of neutrophil infiltrate in tumor lesions, which is higher in lung squamous cell carcinoma when compared to adenocarcinoma. Furthermore, in addition to maintaining a state of chronic inflammation, which by itself is an important risk factor for cancer development, the neutrophils, after entering the cells, produced elastase which may further contribute to the pathogenesis of lung cancer by inducing mitogenesis. Lastly, assessing the neutrophil/lymphocyte ratio (NLR) can provide prognostic value in both small and non-small lung cancers. A high NLR is associated with poor clinical outcome and decreased overall survival rate. In view of the aforementioned, the potential paraneoplastic connection of Sweet syndrome with hematogenous as well as solid tumors should be taken into account and a full work-up for malignancy should be considered, particularly for older individuals with other risk factors [28].* Respiratory comorbidities of Sweet syndrome are summarized in Figure 3.*

#### 2.4.4. Implications of This Association on Treatment Strategies

Although the most common treatment of Sweet syndrome remains systemic steroids, the increasing number of fulminant refractory cases together with the recent advances in the molecular pathogenesis have introduced targeted treatment as a safer, more precise, and effective therapy. Delluc et al. were the first to report a dramatic efficacy of Anakinra in the treatment of a recalcitrant case of Sweet syndrome with alveolar lung condensation [98]. In 2011, Anakinra was successfully used in the treatment of another resistant case. This shed light not only on the promising role of the IL-1 receptor antagonist in the treatment of refractory cases with comorbidities, but also on the importance of the autoinflammation and the inflammasome theory in the etiopathogenesis of Sweet syndrome [99].

### 2.5. Subcorneal Pustular Dermatosis and Associated Respiratory Conditions

#### 2.5.1. Subcorneal Pustular Dermatosis (SPD)

Subcorneal pustular dermatosis (SPD) is a rare chronic relapsing dermatosis, first described by Sneddon and Wilkinson in 1956 [100]. The presence of the following criteria is necessary for diagnosing SPD: new onset symmetrical, flaccid pustular eruption distributed in a gyrate pattern healed with annular shaped scale predominantly involving flexural aspect of trunk and intertriginous regions, histologically characterized by a sterile subcorneal neutrophilic infiltration without spongiosis or acantholysis, and an excellent response to dapsone treatment. Differential diagnosis includes SPD-type IgA pemphigus and pustular psoriasis which can be distinguished by immunological studies. SPD has been reported in association with many extracutaneous disorders including hematogenous conditions such as multiple myeloma, lymphoma, and monoclonal gammopathy, inflammatory bowel disease, and infections like mycoplasma pneumoniae [9, 101–103].

#### 2.5.2. Mycoplasma pneumoniae

*Mycoplasma pneumoniae* mainly affects respiratory airways with the majority of cases being self-limited. It has recently been proven that the severity of pneumonia caused by* M. pneumoniae* depends primarily on innate immunity. Severe infections have a substantial neutrophilic infiltration compared to the recovery stage which showed a marked reduction in the number of polymorphonuclear leucocytes [28, 104]. The clearance of the pathogen is thought to be Th17 driven as opposed to Th1 and Th2 cells, as was demonstrated by Wu et al. IL-17 production is essential in neutrophil recruitment and activity in the lung defense against M. pneumoniae infection, so it is plausible to hypothesize that a dysregulated Th17 immune response in genetically predisposed individuals will result in perpetual recruitment and activation of neutrophils resulting in a severe pneumonia [105].

In addition,* M. pneumonia* is notorious for extrapulmonary involvement in at least 25% of cases with the majority including cutaneous and mucosal manifestations that can sometimes present without any preceding respiratory symptoms. The mechanism of such associations can be explained by cytokine mediated local inflammation or indirect modulation of immune response and deposition of immune complexes [104, 105].

#### 2.5.3. Association of SPD with M. Pneumoniae

Until 2015, nine cases of SPD have been described in association with* M. pneumoniae* respiratory infections.* This has* been related to the indirect effects of the organism on host innate and adaptive cell mediated immunity, potentially through upregulation of neutrophilic chemotaxis and activation in* M. pneumoniae* which triggers the development of SPD [31, 106–113]. Further supporting the role of neutrophils in this association is the dramatic response of SPD to dapsone. Dapsone works through the inhibition of integrin mediated neutrophilic adherence and chemotaxis [102, 114]. Additionally, this association should draw attention to the possibility of* M. pneumoniae* being manifested as pustular and bullous skin conditions. Importance must be given to the screening for the infection in patients presenting with these rare blistering dermatoses and particularly being aware of the benign course of the associated cutaneous condition which does not necessitate systemic treatment [101, 112].

### 2.6. Pustular Psoriasis and Respiratory Comorbidities

#### 2.6.1. Pustular Psoriasis

Pustular psoriasis is an uncommon variant of psoriasis vulgaris, a chronic systemic inflammatory disease [115–117]. It is known for its cutaneous and extracutaneous manifestations, which presents clinically as superficial sterile pustular eruption on erythematous base [118]. Pustular psoriasis can manifest as a localized entity in cases of palmoplantar pustular psoriasis and Acrodermatitis continua of Hallopeau or in a generalized diffuse life-threatening form as seen with infantile and juvenile pustular psoriasis, pustular psoriasis of pregnancy, and generalized pustular psoriasis (GPP). Whether it is localized or generalized, all subtypes of pustular psoriasis share the same histological features. These include parakeratosis, extensive polymorphonuclear inflammatory infiltrate, that can cluster anywhere in the epidermis including stratum corneum, also known as Munro microabscess, spinous zone in association with spongiosis, referred to as spongiform pustules of Kogoj, atrophy of stratum granulosum, and dilated tortuous papillary capillaries responsible for Auspitz sign of psoriasis [119, 120].

#### 2.6.2. Pustular Psoriasis Triggered by URTIs

Upper respiratory tract infections (URTIs), especially streptococcal tonsillitis, are one of the most frequently reported triggers for the development of generalized as well as infantile and juvenile pustular psoriasis variants. This could be related to genetically determined hypersensitivity to microbial antigens [120–122]. In a prospective cohort study including 3994 subjects with psoriasis and 10000 matched healthy controls conducted by Huerta et al. in 2007,* S. pyogenes* was isolated from up to 97% of psoriatic patients aged between 21 and 40 years [123]. In another study performed by Seyhan et al. in 2006, URTIs were reported as the most common predisposing factor of psoriasis in childhood and adolescents [124]. Reported precipitating factors include corticosteroid withdrawal, vaccines, and some antibiotics [120].

**Figure 4:**
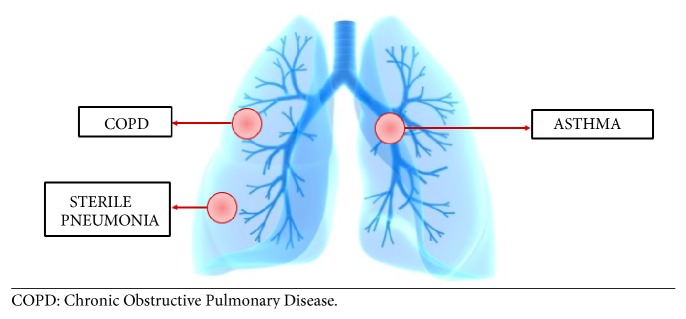
Respiratory comorbidities described in the context of pustular psoriasis.

Pathogenesis of pustular psoriasis is still not fully understood; however recent evidence points to it being an autoinflammatory disorder based on genetic and immunological studies with a hallmark of overexpression of IL-1 cytokines or disinhibition of their signaling pathway [125]. The genetic deficiency of IL-36 receptor antagonist (IL-36RN), also referred to as DITRA recently identified in all familial cases and few sporadic cases of GPP, has further supported this hypothesis. IL-36RN, predominantly expressed in skin, acts to antagonize three IL-36 cytokines, all of which belong to the proinflammatory IL-1 family, the main effector molecule in the pathogenesis of autoinflammation [126].* Respiratory comorbidities described in pustular psoriatic patients are summarized in Figure 4.*

#### 2.6.3. Pustular Psoriasis and Sterile Pneumonitis

Remarkably, subclinical inflammation in psoriatic patients, without respiratory diagnosed comorbidities, [127, 128] was highlighted recently by the use of fraction exhaled nitric oxide, a noninvasive tool routinely used in diagnosing and monitoring asthma and COPD [129–131]. Although rare, pulmonary complications described with pustular psoriasis can be life threatening and therefore require awareness and immediate intervention [132–134]. Among these fulminant conditions is the sterile pneumonitis, a respiratory noninfectious acute respiratory distress syndrome associated with GPP, first described by Landry and Muller in 1972 [135]. Until 2011, thirteen cases [99, 135–142] were described in the literature. All cases presented with a rapidly progressive deterioration in respiratory functions, manifested as dyspnea and severe hypoxemia that were preceded by acute flare of their cutaneous condition. Another cardinal feature of this association was the significantly high level of neutrophilia reported in all patients. Imaging studies including chest X-ray and CT scan with contrast revealed bilateral interstitial and alveolar infiltrate. The condition was differentiated pneumonia and congestive heart failure caused by the lack of effectiveness of empirical broad spectrum antibiotics and loop diuretics, respectively. In addition hypersensitivity to commonly prescribed psoriasis medications was also excluded. The outcome was generally bad except for those who immediately initiated on a high dose of corticosteroids. As an explanation of this association, cases' reporters have postulated a role of high level of circulating proinflammatory cytokines in particular TNF-alpha in the massive recruitment of mononuclear and polymorphonuclear cells with a resultant extensive alveolar inflammation and capillary leak [99].

#### 2.6.4. Psoriasis and COPD

A population-based case control study conducted by Dreiher et al. in 2008 investigated the association between psoriasis and COPD [143]. According to the authors, the prevalence of COPD was significantly higher among psoriatic subjects compared to controls. Additionally, two meta-analyses have recently reviewed the correlation between psoriasis and COPD [144, 145]. According to both meta-analyses there was a significant higher susceptibility to COPD in psoriatic patients when compared to controls. The mechanism of this association was explained by the fact of both conditions being chronic inflammatory diseases with an imbalance between the proinflammatory activity of Th17 and the anti-inflammatory role of Treg with an overexpression of IL-17A and IL-17F in both conditions. IL-17 is known to trigger and amplify a neutrophilic mediated immune response. Also, of note, IL-36, one of the cardinal psoriatic cytokines, has been found to be elevated in the COPD patients' lavage. Furthermore, a defect in the signals controlling polymorphonuclear chemoattraction and elastase release was linked to mucin hypersecretion and a compromised lung function. Consistent with this explanation was the finding of an excessive level of proinflammatory cytokines such as TNF-alpha, IL-6, and IL-8 in circulation. In view of this association, dermatologists should advise their patients about the importance of smoking cessation and the avoidance of other risk factors that can further positively feed this inflammation loop [143, 146].

#### 2.6.5. Psoriasis and Asthma

Only few studies have highlighted the increased risk of developing asthma in psoriatic patients. A population-based retrospective cohort study with 10288 recruited psoriatic subjects has revealed a significant increase risk of asthma in patients suffering from psoriasis [147]. A meta-analysis performed recently by Wang et al., including a total number of 66,772 patients with psoriasis, has shown an increased susceptibility of asthma among these patients [148]. Asthma is now considered a general term and defined as a state of airway hyperresponsiveness with subsequent inflammation and reversible obstruction. This umbrella includes many entities depending on the predominant leukocytic infiltrates. Dysregulated activity of Th17 cells, mediated by proinflammatory cytokines such as IL-1*β*, IL-6, IL-23, and TGF*β*, was proposed as a crucial trigger and amplifier of both cutaneous and airway inflammation especially neutrophilic asthma, a hypothesis further supported by the efficacy of blocking Th17 cytokines such as IL-12/IL-23 in controlling both conditions, in particular those that were not responsive to conventional therapy [148–150].

## 3. Conclusion

Since the first reported case of PG in 1908, research unraveling the etiopathogenesis of neutrophilic dermatoses has continuously evolved. Given the frequent association with extracutaneous manifestations where neutrophils seem to play a crucial role, it is plausible to consider NDs as a cutaneous presentation of a systemic neutrophilic condition. Additionally, accumulating evidence suggests this entity of dermatoses may be part of an autoinflammatory condition, which is supported by the efficacy of IL-1 blockers in management of recalcitrant cases. Moreover, a prominent role of an aberrant adaptive immune reaction with imbalance between proinflammatory Th17 and anti-inflammatory Treg immune responses was described; this was underpinned by the overexpression of cytokines involved in Th17 polarization. In the previous correlations summarized in this review, pulmonary involvement was not uncommonly reported with NDs. Associated respiratory condition can serve to be a trigger, as seen with URTIs frequently preceding the rash of Sweet syndrome and pustular psoriasis. Similarly* M. pneumoniae *infection was frequently reported with the rare entity of SPD so that SPD was referred to as the cutaneous manifestation of* M. pneumoniae*. At other times, respiratory symptoms can develop or be exacerbated secondary to the uncontrolled skin disorder as is the case with asthma and COPD whose risk was significantly higher with pustular psoriasis and HS. Furthermore, life-threatening complications were recounted in association with NDs such as the development of sterile pneumonitis described more than once with pustular psoriasis. Many potential mechanisms were postulated to explain this association. These include aberrant neutrophil chemotaxis and activation that was either connected to a genetic defect in innate immunity such as autoinflammation or tied to a dysregulated adaptive immune response with excessive Th17 polarization or a paraneoplastic phenomenon. In view of the above, it is important to apply this knowledge in the three steps of patients' management, work-up, diagnosis, and treatment. Given the well-established paraneoplastic association, a thorough work-up to rule out hematogenous as well as solid malignancy is crucial in every patient especially when other risk factors coexist. Additionally, while making the diagnosis, it is important to define the chronological order of the conditions and determine whether the ND is the primary event or the consequent condition. This was the case with pulmonary tuberculosis reported with Sweet's syndrome, where therapy was directed toward primarily controlling the respiratory infection before rushing into high doses of corticosteroids to treat the cutaneous disease. Physicians should be aware of the systemic associations of NDs including the respiratory conditions which may redirect their therapeutic strategies either to address both conditions with one treatment or to avoid the exacerbation of one disease while attempting to treat the other [6].

## Figures and Tables

**Table 1:** Pulmonary comorbidities and concurrent pulmonary findings in neutrophilic dermatoses.

NEUTROPHILIC DERMATOSES	PULMONARY COMORBIDITIES	PULMONARY FINDINGS	REFERENCES	NOTES
Hidradenitis Suppurativa	(1) Asthma (2) COPD (3) Interstitial pneumonia	-	(1) Magun et al., 2016. (2) Magun et al., 2016. (3) Damiani et al., 2017.	-

Pyoderma Gangrenosum	(1) Interstitial Infiltrates causing cavitation (2) Pulmonary infiltrates without cavitation	(3) Pulmonary nodules (4) Cystic lung lesions (5) Pleural effusion (6) Airway impairment	(1) Lebbe et al., 1992, Brown et al., 2000, Liu et al., 2008, Vignon-Pennamen et al., 1989, Fukuhura et al., 1998, Kruger et al., 2001, Chahine et al., 2007, Batalla et al., 2011, Bittencourt et al., 2012 (2) Vignon-Pennamen and Wallach, 1991, Urano et al., 1995, Takeuchi et al., 2003, Bhat et al., 2007, Kitagawa et al., 2008, MCCulloch et al. 1985, Shands et al., 1987, Peters et al., 1998, Wang et al., 1999, Field et al., 2008, Rajan et al., 2009, Kanoh et al., 2009 (3) Kasuga et al., 1999, Matsumura et al., 2011 (4) Grattan et al., 1998 (5) Vadillo et al., 1999, Fukuhara et al., 1998, Peters et al., 1998, Kasuga et al., 1999, Wang et al., 1999. (6) Merge et al., 1998	(1)-(6). lung biopsy showed neutrophilic infiltrates

Sweet syndrome	(1) Unilateral interstitial infiltrates and bronchiolitis obliterans (2) Unilateral interstitial infiltrates & pleural effusion (3) Bilateral interstitial infiltrates (4) Bilateral interstitial in- filtrates & pleural effusion	(5) URTIs (6) Interlobular septal thickening (7) Pulmonary tuberculosis (8) Lung cancer (a) Small cell carcinoma (b) SCC adenocarcinoma	(1) Angeline et al., 1986, Takimoto, 1991, Chien, 1991, Kushima et al., 2007, Robbins et al., 2009, Keefe et al., 1998, Reid et al., 1996, Peters et al., 1998, Katsura et al., 1999, Longo et al., 2001, Lawrence et al., 2008, Aparicio, 2010 (2) Fernandez-Bussy et al., 2012, Soderstrom, 1981, Rodriguez de la Serna et al., 1985, Garg et al. 2006. (3) Gibson et al., 1985, Hatch et al., 1989, Bourke et al., 1991, Komiya et al., 1991, Cohen and Kurzrock, 1989, Fett et al., 1995, Rodot et al.,1996, Thurnheer et al., 1997, Alberts, 2000, Imanaga et al., 2000, Petrig et al., 2006, Fulton, 2007, Aydemir, 2008 (4) Fett et al., 1995, Ravaglia et al., 2015 (5) Lallas et al., 2011, Volpe et al., 2016 (6) Astudillo, et al., 2006 (7) Serirat and Thaipisuttikul, 2011, Trabulo et al., 2015, Chauhan et al., 2018 (8) Yamamoto et al., 1994, Denhove et al., 2007, Nielsen et al., 1993, Arai et al., 2008	(1)-(4). Lung biopsy showed neutrophilic infiltrate (8) Associated with cancer cervix or Crohn's disease.

Subcorneal Pustular Dermatosis	-	*M. pneumoniae*	Teisch et al., 1970, Sneddon, 1973, Matsubara et al., 1982, Winnock Et al., 1996, Reichert‐ Penetrat et al., 2000, Papini et al., 2003, Kim et al., 2006, Lombart et al., 2014, Bohelay et al., 2015	

Pustular Psoriasis	(1) COPD (2) Asthma	(3) URTIs (4) Sterile Pneumonitis	(1) Dreiher et al., 2008, Li et al., 2015, Ungprasert et al., 2016 (2) Fang et al., 2015, Wang et al., 2018 (3) Seyhan et al., 2006, Huerta et al., 2007, Speeckaert et al., 2010, Benjegerdes et al., 2016 (4) Landry & Muller, 1972, McGregor et al., 1991, Hand- field-Jones et al., 1992, Donnell et al., 1995, Sadeh et al., 1997, Doval et al., 1998, Abou‐Samra et al., 2004, Griffiths et al., 2006, Kluger et al., 2011	-

COPD: chronic obstructive pulmonary disease, URTIs: upper respiratory tract infections, *M. pneumoniae*: *Mycoplasma pneumoniae*.

**Table 2:** Major and minor criteria for the diagnosis of Sweet syndrome.

Major Criteria
(1) Clinically: sudden eruption of tender erythematous papules, coalescent plaques, or nodules commonly affecting face, neck, and the upper limbs (2) Histologically: extensive dermal neutrophilic infiltrate and edema in the absence of leukocytoclastic vasculitis

Minor Criteria

(1) Fever > 38°C (2) Preceded by vaccination or URTI*∗* or GIT*∗* infections, associated malignancy ( visceral or hematologic) or pregnancy (3) Dramatic response to systemic steroids or potassium iodide (4) Abnormal lab*∗* values at presentation (three of four): ESR*∗* >20 mm/hr; positive CRP*∗*; WBCs*∗* >8,000; >70% neutrophils)

URTI: upper respiratory tract infections, GIT: gastrointestinal tract, ESR: erythrocyte sedimentation rate, CRP: C reactive protein, WBCs: white blood cells.
